# Crystal structure and Hirshfeld surface analysis of *catena*-poly[4-amino-4*H*-1,2,4-triazol-1-ium [lead(II)-tri-μ-bromido]]

**DOI:** 10.1107/S2056989025009557

**Published:** 2025-11-06

**Authors:** Olesia I. Kucheriv, Sergii O. Malinkin, Olena Prysiazhna, Alexandru Constantin Stoica, Irina A. Golenya

**Affiliations:** a Department of Chemistry, Taras Shevchenko National University of Kyiv, Volodymyrska St. 64, 01601 Kyiv, Ukraine; b Bakul Institute for Superhard Materials, National Academy of Sciences of Ukraine Avtozavodskaya St 2 Kyiv 04074 Ukraine; c Department of Chemistry, Kyiv National University of Construction and Architecture, Povitroflotsky Ave. 31, Kyiv 03680, Ukraine; d "Petru Poni" Institute of Macromolecular Chemistry, Aleea Gr. Ghica Voda 41A, 700487 Iasi, Romania; University of Neuchâtel, Switzerland

**Keywords:** crystal structure, organic–inorganic hybrids, one-dimensional polymeric chain, lead, triazolium cation

## Abstract

Hybrid organic—inorganic perovskites are a group of versatile materials with outstanding performance in photovoltaics, LEDs, lasers, and sensors. The hybrid organic–inorganic compound (4-amino-1,2,4-triazolium)PbBr_3_, or {(C_2_H_5_N_4_)[PbBr_3_])_*n*_, crystallizes in the polar ortho­rhom­bic space group *Pna*2_1_. Its structure is built from [PbBr_6_] octa­hedra with pronounced trigonal distortion, which are connected through face-sharing to form infinite one-dimensional chains extending along the *c-*axis direction. These inorganic chains are separated by 4-amino-1,2,4-triazolium cations that establish an extensive network of weak inter­actions, including N—H⋯Br hydrogen bonds as well as C—H⋯Br contacts and N⋯Pb tetrel bonds. Additionally, N—H⋯N inter­actions link neighboring organic cations. The network of inter­molecular contacts was further examined using Hirshfeld surface analysis and two-dimensional fingerprint plots.

## Abstract 2

The hybrid organic–inorganic compound (4-amino-1,2,4-triazolium)PbBr_3_ crystallizes in a polar space group and features polymeric one-dimensional inorganic chains formed by face-sharing distorted octa­hedra, which alternate with organic cations.

## 1. Chemical context

Organic–inorganic hybrid perovskites have emerged as a highly versatile class of functional materials, displaying exceptional performance in photovoltaics, light-emitting devices, lasers, and sensors (Zhao & Zhu, 2016 ▸). Their appeal arises from the combination of tunable optoelectronic properties, solution-processable fabrication, and structural flexibility that enables a wide spectrum of chemical designs (Younis *et al.*, 2021 ▸). Early research was dominated by three-dimensional perovskites such as CH_3_NH_3_PbI_3_, which exhibit strong light absorption and long carrier diffusion lengths, making them highly efficient in solar energy conversion and photodetection (Quarti *et al.*, 2016 ▸). Nevertheless, the centrosymmetric crystal structures typical of 3D perovskites restrict the emergence of spontaneous polarization, limiting their utility in self-powered photodetectors and bulk photovoltaic effect-based devices (Li *et al.*, 2025 ▸).

To overcome these limitations, considerable attention has been directed toward designing polar hybrid perovskites. The introduction of symmetry-breaking distortions or large organic cations has been shown to stabilize polar structures, thereby enabling spontaneous polarization and associated functionalities (Ji *et al.*, 2019 ▸). Hybrid perovskites, with their adjustable inorganic frameworks and diverse organic cation chemistry, offer an attractive alternative route to engineer polar semiconductors with more favorable bandgaps and carrier dynamics (Xu *et al.*, 2019 ▸).

So-called low-dimensional perovskites have been particularly useful in tailoring polar structures. Two-dimensional perovskites incorporating bulky or chiral organic cations can adopt non-centrosymmetric lattices that support ferroelectricity and intrinsic bulk photovoltaic effect (Li *et al.*, 2021 ▸). Moreover, their structural distortions can induce broadband white-light emission *via* self-trapped excitons, a feature that has been linked to strong electron–phonon coupling in corrugated inorganic frameworks (Wang *et al.*, 2018 ▸). Such multifunctionality highlights the inter­play between lattice distortion, optical properties, and polarity in hybrid perovskites, and it demonstrates their promise as candidates for next-generation optoelectronic devices.
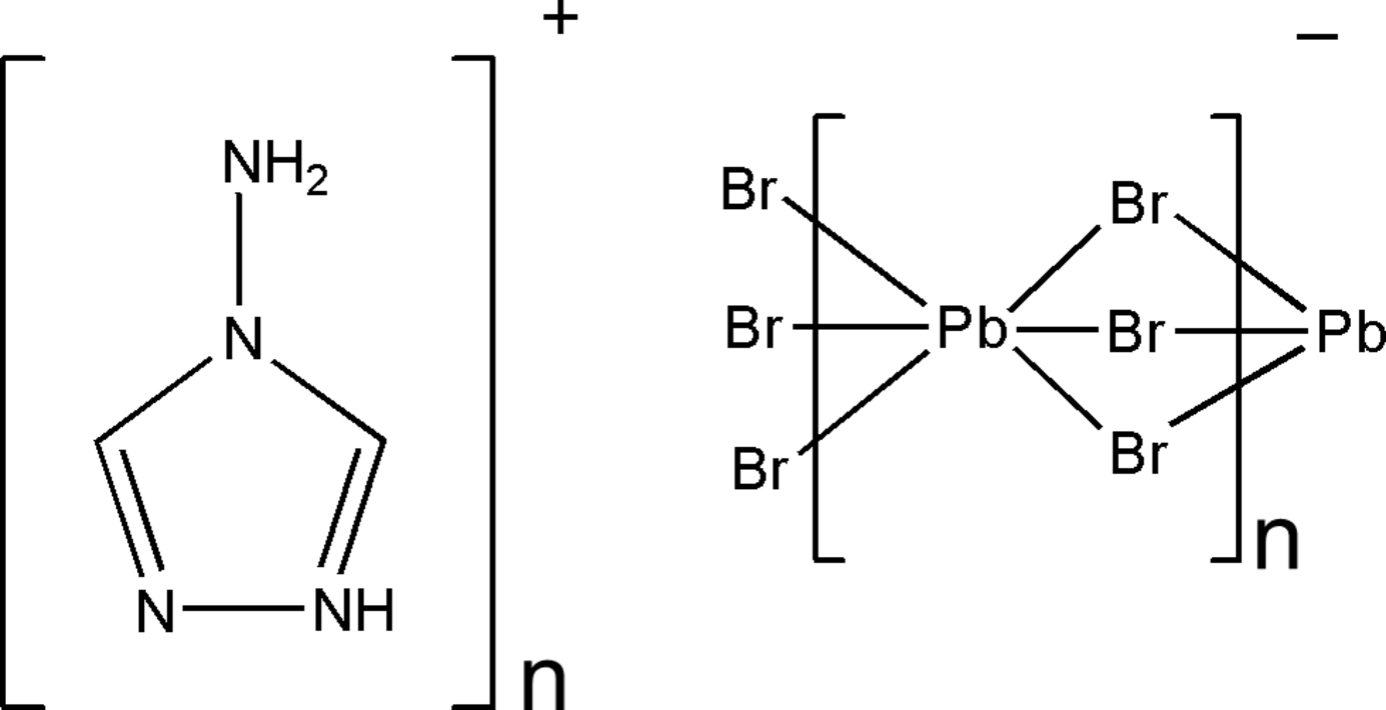


Taken together, these developments underscore the importance of polarity in hybrid perovskites for enabling novel optoelectronic phenomena and device concepts. Rational design strategies, whether through dimensional reduction, chiral templating, or cation substitution, continue to expand the library of polar perovskites with tailored bandgaps and multifunctional properties. In this context, crystallographic investigations of new polar hybrid perovskites are crucial, as they provide the structural insights necessary to understand structure–property relationships and to guide further material design. The present work contributes to this effort by reporting and analyzing the crystal structure of a new polar hybrid organic–inorganic compound (4-amino-1,2,4-triazolium)PbBr_3_.

## 2. Structural commentary

**Figure 1:**
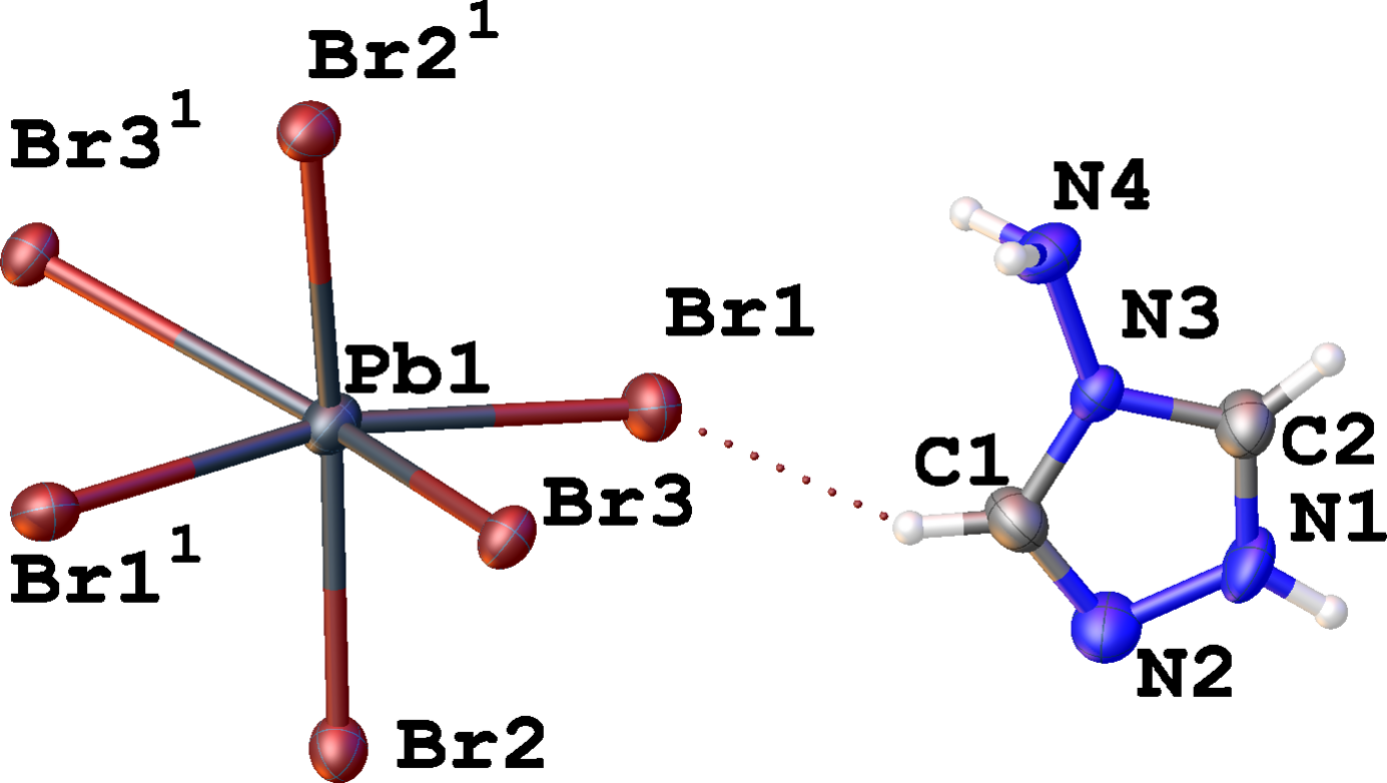
Fragments of (4-amino-1,2,4-triazolium)PbBr_3_ showing the atom-labeling scheme, and a strong inter­action between 4-amino-1,2,4-triazolium and the PbBr_6_ octa­hedron (dotted line). Displacement ellipsoids are drawn at the 50% probability level. [Symmetry code: (1) 1 − *x*, 1 − *y*, −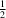 + *z*]

**Figure 2:**
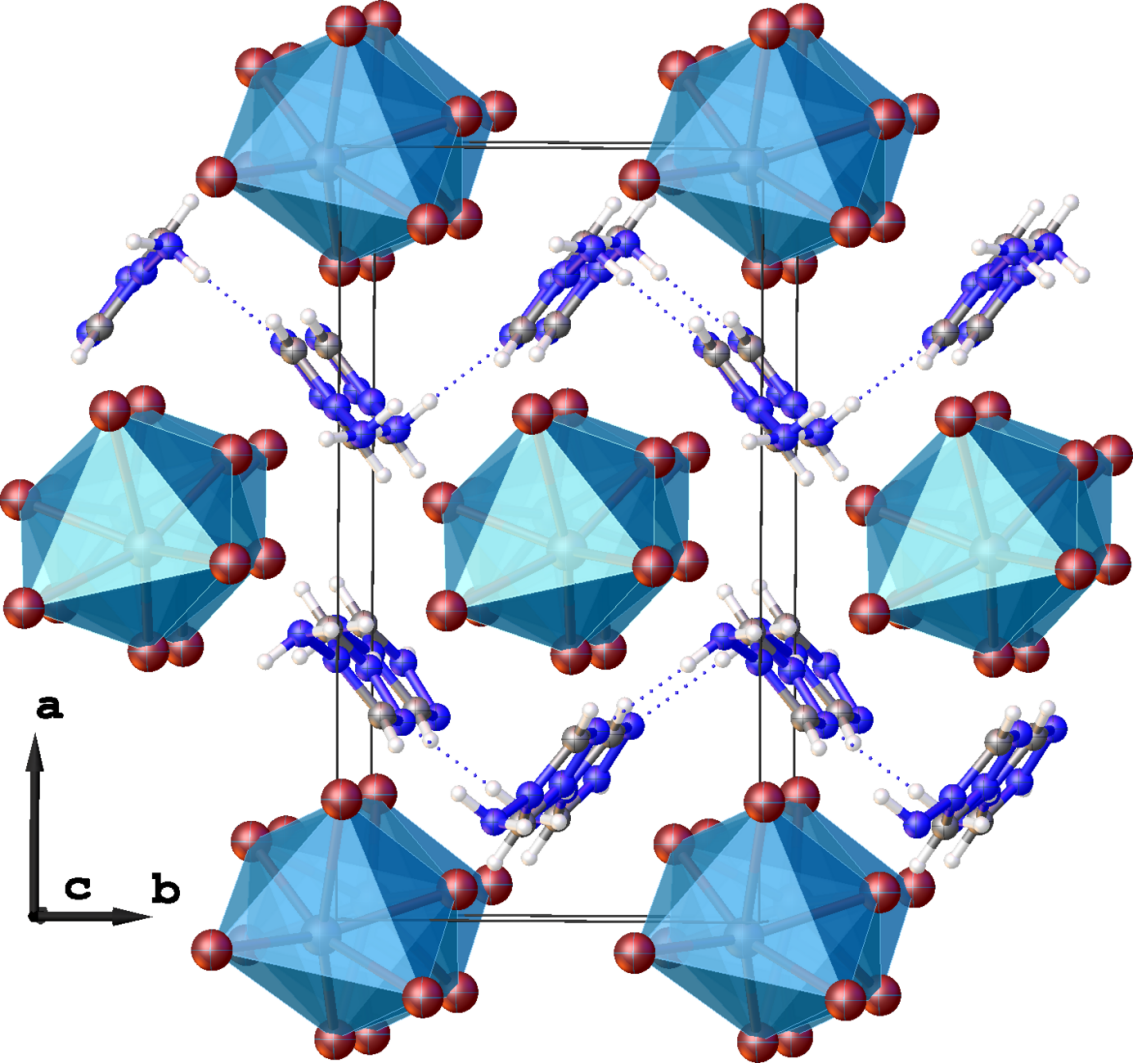
Fragment of the crystal structure of (4-amino-1,2,4-triazolium)PbBr_3_ showing the propagation of the infinite one-dimensional face-shared chains along the *c-*axis direction. N—H⋯N hydrogen bonds are shown as blue dotted lines.

The title compound crystallizes in the non-centrosymmetric space group *Pna*2_1_. In this crystal structure, Pb^2+^ exhibits an octa­hedral coordination environment provided by six bromide anions, which features significant trigonal distortion (Fig. 1 ▸). The inorganic [PbBr_6_] octa­hedra connect with each other in face-sharing manner creating infinite 1D chains which propagate along the *c-*axis direction (Fig. 2 ▸). The creation of similar faced-shared 1D chains has been previously observed for organic–inorganic hybrids with substituted imidazolium cations (Thirumurugan & Rao, 2008 ▸; Kobayashi *et al.*, 1972 ▸). The Pb—Br bond lengths are in the in the range 2.9200 (8) to 3.2563 (9) Å, the observed octa­hedral distortion can be qu­anti­tatively estimated by quadratic elongation parameter: <λ_oct_> = Σ(*l*_i_/*l*_0_)^2^/6 = 0.013, where *l*_i_ are six Pb—Br bond lengths and *l*_0_ is the average Pb—Br bond length (Robinson *et al.*, 1971 ▸). The σ_θ_^2^ = Σ(θ_i_ – 90)^2^/11 = 237.72, where θ_i_ are twelve *cis*-Br—Pb—Br angles (Robinson *et al.*, 1971 ▸). Such a large deviation of *cis*-Br—Pb—Br angles and consequent large bond-angle variance is not very common for lead halides, though not unique, and has previously been observed for compounds that form similar face-shared 1D inorganic chains (He *et al.*, 2019 ▸; Tang & Guloy, 1999 ▸).

The inorganic 1D chains are separated by 4-amino-1,2,4-triazolium organic cations, which compensate the negative charge of the inorganic component. All bond lengths and angles in this organic cation are within the expected range (Allen *et al.*, 1987 ▸).

## 3. Supra­molecular features

**Figure 3:**
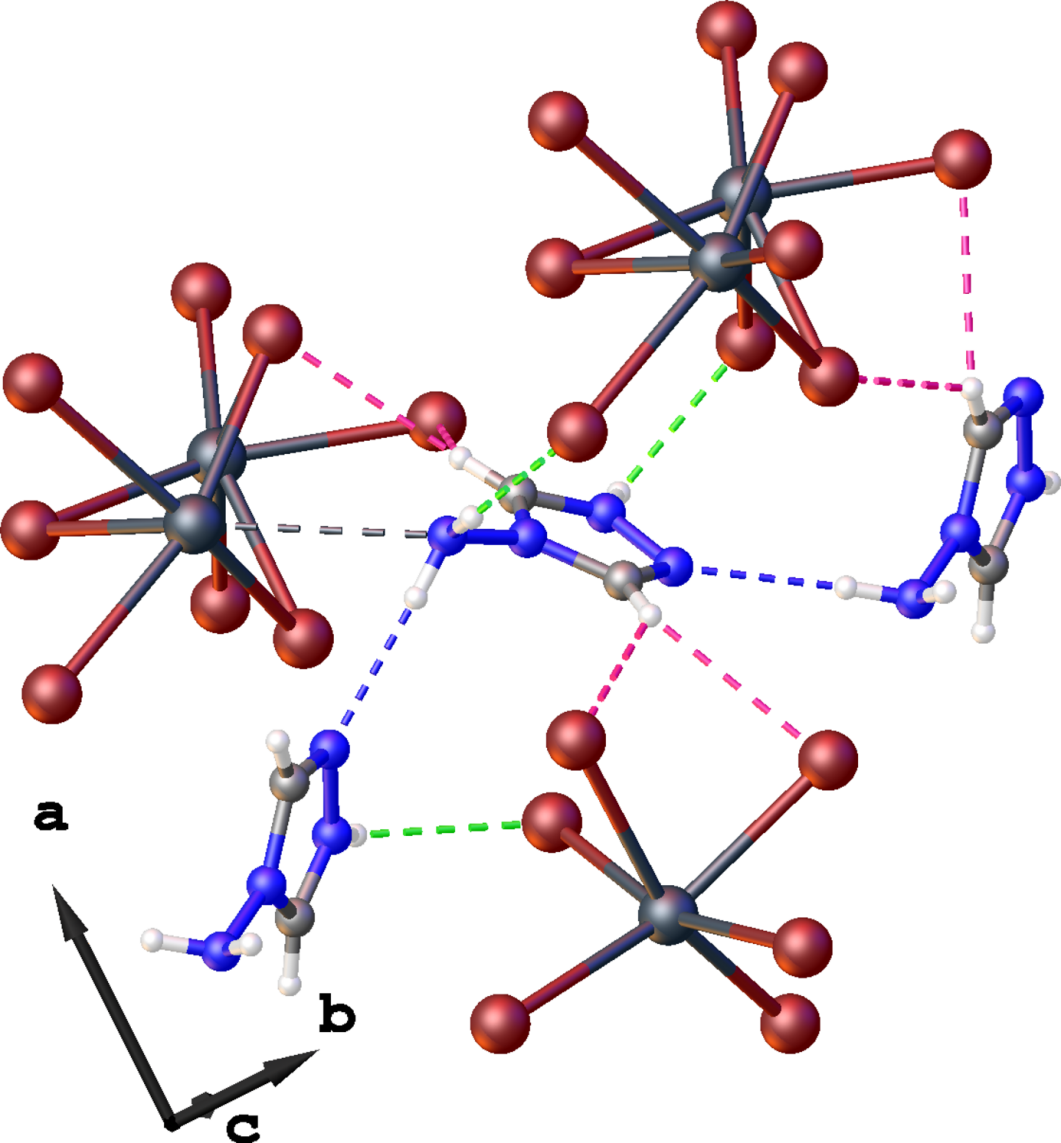
Weak inter­actions present in the structure: tetrel N⋯Pb bond (black dashed lines), N—H⋯Br (green dashed lines), N—H⋯N (blue dashed lines) and C—H⋯Br (pink dashed lines).

The organic cations inter­act with the inorganic 1D chains by a network of weak inter­actions (Fig. 3 ▸). The amino group participates in two hydrogen bonds: an N4—H4*B*⋯N2^ii^ [symmetry code: (ii) −*x* + 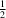, *y* − 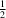, *z* − 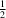] contact with a neighboring amino­triazolium cation, and an N4—H4*B*⋯Br1 contact with a bromine from a neighboring inorganic polymeric chain. Detailed geometry of these hydrogen bonds can be found in Table 1 ▸. In addition, four relatively short C—H⋯Br contacts (all H⋯Br < 3.0 Å) that help consolidate the packing are observed. In addition, the Pb1⋯N4 distance is 3.357 (7) Å, which is significantly shorter than the sum of the van der Waals radii of the corresponding elements (4.2 Å). This short contact can be inter­preted as a non-covalent tetrel bond, in which the lead atom acts as a tetrel-bond donor possessing an electrophilic region on its surface, while the nitro­gen atom serves as a nucleophilic tetrel-bond acceptor with an electron pair (Varadwaj *et al.*, 2023 ▸; Scheiner, 2021 ▸).

## 4. Hirshfeld surface analysis

**Figure 4:**
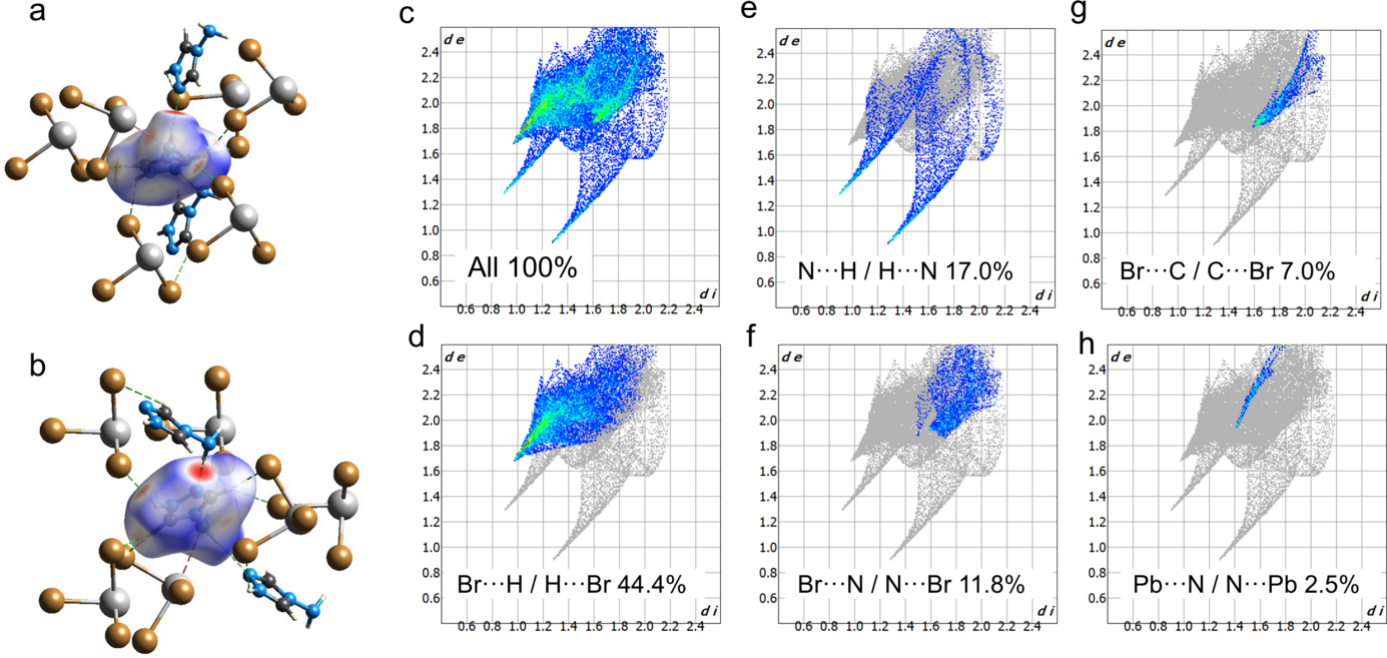
(*a*),(*b*) Hirshfeld surface highlighting the strength and distribution of inter­molecular inter­actions between the organic and inorganic components of the title compound. (*c*)–(*h*) The corresponding fingerprint plots illustrating the frequency of specific inter­molecular contacts within the crystal structure.

Inter­molecular inter­actions in the title compound were additionally analyzed using Hirshfeld surface and fingerprint plots obtained with *CrystalExplorer* (Spackman *et al.*, 2021 ▸). To visualize inter­molecular inter­actions, the Hirshfeld surface was plotted with *d*_norm_ at the conventional resolution and rendered with a fixed color scheme (Fig. 4 ▸*a*–*b*): regions where inter­atomic separations approximate the sum of van der Waals radii are depicted in white, shorter contacts are highlighted in red, and longer ones in blue. The fingerprint plots depict how often these inter­actions appear in the crystal structure. Hence, the Hirshfeld surface and the 2D plots convey different aspects: one reflects contact strength, the other their frequency. The red regions of the Hirshfeld surface here mostly correspond to stronger N—H⋯Br and N—H⋯N contacts, while pale pink regions can be observed for —H⋯Br and Pb⋯N inter­actions.

The two-dimensional fingerprint plots (Fig. 4 ▸*c*–*h*) show that the most frequently observed meaningful weak inter­actions in the structure are Br⋯H/H⋯Br contacts, which make a 44.4% contribution to the overall number of inter­actions. Other contacts that make notable contributions include Br⋯N/N⋯Br (11.8%) and N⋯H/H⋯N (17.0%). Br⋯C/C⋯Br and Pb⋯N/N⋯Pb make 7.0 and 2.5% contributions, respectively. The observed Br⋯C/C⋯Br contact can be attributed to a shifted weak π⋯Br inter­action oriented toward the C atom of the triazole ring [Br2⋯C1 = 3.487 (8) Å, ring centroid⋯C—Br = 96.8 (4)°]. The remaining inter­actions are H⋯H contacts, which occur frequently in the structure as a result of the terminal hydrogen-atom positions; nevertheless, they lack chemical significance.

## 5. Database survey

A survey of the Cambridge Structural Database (CSD version 5.45, update of September 2024; Groom *et al.*, 2016 ▸) revealed that the formation of organic–inorganic compounds with [PbBr_6_]^4−^ octa­hedra that combine in a face-sharing manner is quite common (101 hits). It is specifically worth paying attention to (3-amino-1,2,4-triazolato)PbBr_3_, which is isostructural with the title compound (Li *et al.*, 2007 ▸). The 4-amino-1,2,4-triazolium cation has already been used for the formation of the organic–inorganic hybrid compound bis­(4-amino-1,2,4-triazolium) hexa­chlorido­stannate(IV) (Daszkiewicz & Marchewka, 2012 ▸).

## 6. Synthesis and crystallization

PbBr_2_ (18.3 mg, 0.05 mmol) was dissolved in 0.2 ml of conc. HBr (48%). Then, 4-amino-1,2,4-triazole (21.0 mg, 0.25 mmol) was added to the former solution. Colorless crystals formed on the bottom of the vial within 24 h and were stored in the mother solution prior to SXRD analysis.

## 7. Refinement

Crystal data, data collection and structure refinement details are summarized in Table 2 ▸. H atoms were placed at calculated positions and refined isotropically with *U*_iso_(H) = 1.2*U*_eq_(C) or 1.2*U*_eq_(N). H atoms of the aromatic ring were placed on the external bis­ector of the *X*—C—*Y* or *X*—N—*Y* angle and refined as riding. The H atoms of the amino group were positioned with an idealized geometry (NH_2_, hydrogens lying in the plane of the nearest substituent) and refined as riding.

## Supplementary Material

Crystal structure: contains datablock(s) I. DOI: 10.1107/S2056989025009557/tx2105sup1.cif

Structure factors: contains datablock(s) I. DOI: 10.1107/S2056989025009557/tx2105Isup2.hkl

CCDC reference: 2498871

Additional supporting information:  crystallographic information; 3D view; checkCIF report

## Figures and Tables

**Table 1:** Hydrogen-bond geometry (Å, °)

*D*—H⋯*A*	*D*—H	H⋯*A*	*D*⋯*A*	*D*—H⋯*A*
N4—H4*A*⋯Br2^i^	0.90	2.82	3.700 (7)	167
N4—H4*B*⋯Br1	0.91	3.39	3.763 (6)	107
N4—H4*B*⋯N2^ii^	0.91	2.30	3.203 (9)	177
N1—H1⋯Br2^iii^	0.86	2.76	3.414 (7)	134
C1—H1*A*⋯Br3^iv^	0.93	2.96	3.569 (7)	124
C1—H1*A*⋯Br1	0.93	2.96	3.711 (8)	139
C2—H2⋯Br3^v^	0.93	2.85	3.424 (8)	121
C2—H2⋯Br2^vi^	0.93	2.93	3.774 (8)	152

Symmetry codes: (i) 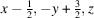; (ii) 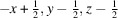; (iii) 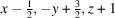; (iv) 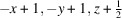; (v) 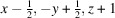; (vi) 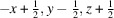.

**Table 2:** Experimental details

Crystal data	
Chemical formula	(C_2_H_5_N_4_)[PbBr_3_]
*M*_r_	532.02
Crystal system, space group	Orthorhombic, *Pna*2_1_
Temperature (K)	293
*a*, *b*, *c* (Å)	14.4941 (3), 7.9506 (2), 8.0569 (2)
*V* (Å^3^)	928.45 (4)
*Z*	4
Radiation type	Mo *K*α
μ (mm^−1^)	31.02
Crystal size (mm)	0.26 × 0.13 × 0.09

Data collection	
Diffractometer	XtaLAB Synergy, Dualflex, HyPix
Absorption correction	Analytical (*CrysAlis PRO*; Rigaku OD, 2024 ▸)
*T*_min_, *T*_max_	0.022, 0.149
No. of measured, independent and observed [*I* > 2σ(*I*)] reflections	11100, 2209, 2054
*R*_int_	0.043
(sin θ/λ)_max_ (Å^−1^)	0.709

Refinement	
*R*[*F*^2^ > 2σ(*F*^2^)], *wR*(*F*^2^), *S*	0.022, 0.047, 1.03
No. of reflections	2209
No. of parameters	92
No. of restraints	1
H-atom treatment	H-atom parameters constrained
Δρ_max_, Δρ_min_ (e Å^−3^)	1.08, −1.00
Absolute structure	Flack *x* determined using 769 quotients [(*I*^+^)−(*I*^−^)]/[(*I*^+^)+(*I*^−^)] (Parsons *et al.*, 2013 ▸)
Absolute structure parameter	−0.025 (7)

Computer programs: *CrysAlis PRO* (Rigaku OD, 2024 ▸), *SHELXT2018/2* (Sheldrick, 2015*a* ▸), *SHELXL2019/3* (Sheldrick, 2015*b* ▸) and *OLEX2* (Dolomanov *et al.*, 2009 ▸).

## supplementary crystallographic information

### catena-Poly[4-amino-4H-1,2,4-triazol-1-ium [lead(II)-tri-µ-bromido]] Crystal data

**Table:**

(C_2_H_5_N_4_)[PbBr_3_]	*D*_x_ = 3.806 Mg m^−^^3^
*M**_r_* = 532.02	Mo *K*α radiation, λ = 0.71073 Å
Orthorhombic, *P**n**a*2_1_	Cell parameters from 7167 reflections
*a* = 14.4941 (3) Å	θ = 2.8–29.6°
*b* = 7.9506 (2) Å	µ = 31.02 mm^−^^1^
*c* = 8.0569 (2) Å	*T* = 293 K
*V* = 928.45 (4) Å^3^	Prism, clear light colourless
*Z* = 4	0.26 × 0.13 × 0.09 mm
*F*(000) = 928	

### catena-Poly[4-amino-4H-1,2,4-triazol-1-ium [lead(II)-tri-µ-bromido]] Data collection

**Table:**

XtaLAB Synergy, Dualflex, HyPix diffractometer	2209 independent reflections
Radiation source: micro-focus sealed X-ray tube, PhotonJet (Mo) X-ray Source	2054 reflections with *I* > 2σ(*I*)
Mirror monochromator	*R*_int_ = 0.043
Detector resolution: 10.0000 pixels mm^-1^	θ_max_ = 30.3°, θ_min_ = 2.8°
ω scans	*h* = −18→20
Absorption correction: analytical (CrysAlisPro; Rigaku OD, 2024)	*k* = −9→10
*T*_min_ = 0.022, *T*_max_ = 0.149	*l* = −10→10
11100 measured reflections	

### catena-Poly[4-amino-4H-1,2,4-triazol-1-ium [lead(II)-tri-µ-bromido]] Refinement

**Table:**

Refinement on *F*^2^	H-atom parameters constrained
Least-squares matrix: full	*w* = 1/[σ^2^(*F*_o_^2^) + (0.0251*P*)^2^] where *P* = (*F*_o_^2^ + 2*F*_c_^2^)/3
*R*[*F*^2^ > 2σ(*F*^2^)] = 0.022	(Δ/σ)_max_ = 0.001
*wR*(*F*^2^) = 0.047	Δρ_max_ = 1.08 e Å^−^^3^
*S* = 1.03	Δρ_min_ = −1.00 e Å^−^^3^
2209 reflections	Extinction correction: SHELXL-2019/2 (Sheldrick 2015b), Fc^*^=kFc[1+0.001xFc^2^λ^3^/sin(2θ)]^-1/4^
92 parameters	Extinction coefficient: 0.0094 (3)
1 restraint	Absolute structure: Flack *x* determined using 769 quotients [(*I*^+^)-(*I*^-^)]/[(*I*^+^)+(*I*^-^)] (Parsons *et al.*, 2013)
Primary atom site location: dual	Absolute structure parameter: −0.025 (7)
Hydrogen site location: mixed	

### catena-Poly[4-amino-4H-1,2,4-triazol-1-ium [lead(II)-tri-µ-bromido]] Special details

**Table:**

Geometry. All esds (except the esd in the dihedral angle between two l.s. planes) are estimated using the full covariance matrix. The cell esds are taken into account individually in the estimation of esds in distances, angles and torsion angles; correlations between esds in cell parameters are only used when they are defined by crystal symmetry. An approximate (isotropic) treatment of cell esds is used for estimating esds involving l.s. planes.

### catena-Poly[4-amino-4H-1,2,4-triazol-1-ium [lead(II)-tri-µ-bromido]] Fractional atomic coordinates and isotropic or equivalent isotropic displacement parameters (Å^2^)

**Table:**

	*x*	*y*	*z*	*U*_iso_*/*U*_eq_	
Pb1	0.47953 (2)	0.53836 (3)	0.04097 (4)	0.03289 (11)	
Br3	0.53855 (5)	0.23101 (10)	0.20857 (10)	0.02956 (17)	
Br2	0.59827 (5)	0.72427 (10)	0.28638 (10)	0.03375 (18)	
Br1	0.34324 (5)	0.52673 (11)	0.32075 (11)	0.0375 (2)	
N3	0.1648 (4)	0.4808 (8)	0.7102 (8)	0.0249 (12)	
N4	0.1308 (4)	0.4089 (9)	0.5628 (9)	0.0344 (14)	
H4A	0.125107	0.485825	0.481111	0.041*	
H4B	0.167219	0.328072	0.517335	0.041*	
N1	0.1775 (5)	0.5361 (9)	0.9650 (9)	0.0386 (16)	
H1	0.167398	0.537000	1.070146	0.046*	
N2	0.2468 (4)	0.6217 (10)	0.8918 (8)	0.0408 (17)	
C1	0.2371 (5)	0.5858 (11)	0.7359 (10)	0.0375 (18)	
H1A	0.275035	0.627032	0.652065	0.045*	
C2	0.1270 (6)	0.4510 (10)	0.8579 (10)	0.0356 (18)	
H2	0.075805	0.384404	0.880651	0.043*	

### catena-Poly[4-amino-4H-1,2,4-triazol-1-ium [lead(II)-tri-µ-bromido]] Atomic displacement parameters (Å^2^)

**Table:**

	*U*^11^	*U*^22^	*U*^33^	*U*^12^	*U*^13^	*U*^23^
Pb1	0.04173 (17)	0.03125 (16)	0.02568 (15)	−0.00392 (10)	−0.00250 (14)	0.00146 (16)
Br3	0.0395 (3)	0.0267 (4)	0.0225 (3)	0.0010 (3)	−0.0002 (3)	0.0003 (3)
Br2	0.0404 (4)	0.0298 (4)	0.0311 (4)	−0.0070 (3)	0.0042 (3)	−0.0012 (3)
Br1	0.0335 (4)	0.0457 (5)	0.0333 (4)	−0.0013 (3)	0.0056 (3)	−0.0005 (4)
N3	0.028 (3)	0.027 (3)	0.020 (3)	0.003 (2)	0.001 (2)	0.000 (2)
N4	0.044 (3)	0.034 (3)	0.026 (4)	−0.001 (3)	−0.005 (3)	−0.005 (3)
N1	0.057 (4)	0.040 (5)	0.019 (3)	−0.005 (3)	0.008 (3)	−0.002 (3)
N2	0.051 (4)	0.035 (5)	0.037 (4)	−0.008 (3)	0.001 (3)	−0.007 (3)
C1	0.033 (4)	0.040 (5)	0.039 (5)	−0.009 (3)	0.004 (3)	−0.004 (4)
C2	0.041 (4)	0.036 (5)	0.030 (4)	−0.005 (3)	0.006 (3)	−0.005 (3)

### catena-Poly[4-amino-4H-1,2,4-triazol-1-ium [lead(II)-tri-µ-bromido]] Geometric parameters (Å, º)

**Table:**

Pb1—Br3	2.9200 (8)	N4—H4A	0.9022
Pb1—Br2	3.0094 (8)	N4—H4B	0.9082
Pb1—Br2^i^	3.1367 (8)	N1—H1	0.8600
Pb1—Br1^i^	3.1646 (9)	N1—N2	1.348 (10)
Pb1—Br1	2.9987 (8)	N1—C2	1.319 (11)
N3—N4	1.407 (9)	N2—C1	1.296 (10)
N3—C1	1.356 (10)	C1—H1A	0.9300
N3—C2	1.332 (11)	C2—H2	0.9300
			
Br3—Pb1—Br2^i^	81.41 (2)	C2—N3—C1	106.9 (7)
Br3—Pb1—Br2	86.54 (2)	N3—N4—H4A	111.8
Br3—Pb1—Br1^i^	83.37 (2)	N3—N4—H4B	115.1
Br3—Pb1—Br1	79.61 (2)	H4A—N4—H4B	103.8
Br2—Pb1—Br2^i^	164.197 (10)	N2—N1—H1	123.6
Br2^i^—Pb1—Br1^i^	79.41 (2)	C2—N1—H1	123.6
Br2—Pb1—Br1^i^	89.11 (2)	C2—N1—N2	112.7 (7)
Br1—Pb1—Br2^i^	103.55 (2)	C1—N2—N1	103.5 (6)
Br1—Pb1—Br2	84.14 (2)	N3—C1—H1A	124.2
Br1—Pb1—Br1^i^	162.02 (2)	N2—C1—N3	111.5 (7)
Pb1—Br3—Pb1^ii^	83.426 (19)	N2—C1—H1A	124.2
Pb1—Br2—Pb1^ii^	84.095 (18)	N3—C2—H2	127.3
Pb1—Br1—Pb1^ii^	83.786 (18)	N1—C2—N3	105.4 (7)
C1—N3—N4	130.5 (6)	N1—C2—H2	127.3
C2—N3—N4	122.5 (6)		
			
N4—N3—C1—N2	−179.0 (7)	C1—N3—C2—N1	−0.7 (9)
N4—N3—C2—N1	179.0 (7)	C2—N3—C1—N2	0.6 (10)
N1—N2—C1—N3	−0.3 (10)	C2—N1—N2—C1	−0.1 (10)
N2—N1—C2—N3	0.5 (9)		

Symmetry codes: (i) −*x*+1, −*y*+1, *z*−1/2; (ii) −*x*+1, −*y*+1, *z*+1/2.

### catena-Poly[4-amino-4H-1,2,4-triazol-1-ium [lead(II)-tri-µ-bromido]] Hydrogen-bond geometry (Å, º)

**Table:**

*D*—H···*A*	*D*—H	H···*A*	*D*···*A*	*D*—H···*A*
N4—H4*A*···Br2^iii^	0.90	2.82	3.700 (7)	167
N4—H4*B*···Br1	0.91	3.39	3.763 (6)	107
N4—H4*B*···N2^iv^	0.91	2.30	3.203 (9)	177
N1—H1···Br2^v^	0.86	2.76	3.414 (7)	134
C1—H1*A*···Br3^ii^	0.93	2.96	3.569 (7)	124
C1—H1*A*···Br1	0.93	2.96	3.711 (8)	139
C2—H2···Br3^vi^	0.93	2.85	3.424 (8)	121
C2—H2···Br2^vii^	0.93	2.93	3.774 (8)	152

Symmetry codes: (ii) −*x*+1, −*y*+1, *z*+1/2; (iii) *x*−1/2, −*y*+3/2, *z*; (iv) −*x*+1/2, *y*−1/2, *z*−1/2; (v) *x*−1/2, −*y*+3/2, *z*+1; (vi) *x*−1/2, −*y*+1/2, *z*+1; (vii) −*x*+1/2, *y*−1/2, *z*+1/2.
